# Liver Steatosis in Induced Hepatocytes From Carriers of Spinal Muscular Atrophy

**DOI:** 10.1002/mus.70111

**Published:** 2026-01-22

**Authors:** Lingyu Sun, Damien Meng Kiat Leow, Loo Chien Wang, Michelle Yating Eio, Hiromi W. L. Koh, Zi Jian Khong, Gunaseelan Narayanan, Aloysius Kai Soon Teo, Richard Giadone, Radoslaw M. Sobota, Shi Yan Ng, Adrian Kee Keong Teo, Wei Yi Ong, Lee L. Rubin, Basil T. Darras, Crystal J. J. Yeo

**Affiliations:** ^1^ Yong Loo Lin School of Medicine National University of Singapore Singapore Singapore; ^2^ National Neuroscience Institute Singapore Singapore; ^3^ Neurobiology Programme, Centre for Life Sciences National University of Singapore Singapore Singapore; ^4^ Bioprocessing Technology Institute (BTI), Agency for Science, Technology and Research (A*STAR) Singapore Singapore; ^5^ Institute of Molecular and Cell Biology (IMCB), Agency for Science, Technology and Research (A*STAR) Singapore Singapore; ^6^ Duke‐NUS Medical School Singapore Singapore; ^7^ Department of Stem Cell and Regenerative Biology Harvard University Cambridge Massachusetts USA; ^8^ Department of Neurology, Boston Children's Hospital, Harvard Medical School Boston Massachusetts USA; ^9^ Lee Kong Chian School of Medicine Nanyang Technological University Singapore Singapore; ^10^ School of Medicine, Medical Sciences, and Nutrition University of Aberdeen Aberdeen UK

**Keywords:** liver steatosis, mitochondrial dysfunction, multisystem disorder, spinal muscular atrophy, stem cell

## Abstract

**Introduction/Aims:**

Although classically characterized as a motor neuron disease, spinal muscular atrophy (SMA) is increasingly recognized as a multisystem disorder. We previously showed hepatocyte‐intrinsic steatosis in SMA, raising the question of whether SMA carriers, who are typically asymptomatic, may also exhibit subclinical hepatic abnormalities.

**Methods:**

We generated induced hepatocyte‐like cells (iHeps) from induced pluripotent stem cells (iPSCs) derived from an SMA Type 2 proband, his isogenic wild‐type (Iso‐WT) line, and both carrier parents, comprised of three carrier lines from the father and one from the mother. Steatosis was assessed by Oil Red O staining and image analysis. Survival motor neuron (SMN) expression was evaluated by immunoblotting. Proteotranscriptomic profiling and mitochondrial respiration assays were performed. Risdiplam, an *SMN2* splicing modulator, was used to assess reversibility of observed phenotypes.

**Results:**

SMA and carrier iHeps demonstrated increased lipid accumulation compared to Iso‐WT. Risdiplam reduced steatosis by 65.9% in SMA patient‐derived iHeps and by 43.6% and 56.9% in father‐ and mother carrier–derived iHeps, respectively. Carrier and SMA iHeps exhibited downregulation of genes involved in lipid metabolism and liver function, along with altered expression of lipid‐related proteins. Mitochondrial dysfunction was present only in SMA iHeps. Carrier‐derived induced motor neurons showed normal viability under oxidative stress, consistent with preserved neuromuscular function clinically.

**Discussion:**

Our data reveal hepatocyte‐intrinsic lipid metabolic defects in SMA carriers, partially reversible with risdiplam. These findings suggest subclinical hepatic involvement in carriers and support further investigation into the systemic impact of SMN deficiency.

**Abbreviations**
iHeps: induced pluripotent stem cell–derived hepatocyte‐like cells
iMNs: induced pluripotent stem cell–derived motor neurons
iPSCs: induced pluripotent stem cells
Iso‐WT: isogenic wild‐type
OCR: oxygen consumption rate
ORO: Oil Red O
SMA: spinal muscular atrophy
SMN: survival motor neuron

## 1 Introduction

Spinal muscular atrophy (SMA) is a monogenic disease caused by biallelic survival motor neuron 1 (*SMN1*) pathogenic variants, leading to deficiency of the SMN protein [1]. SMN is a ubiquitously expressed protein that affects RNA splicing and metabolism [2]. Although its primary function is traditionally thought to be related to the nervous system, there is a growing body of evidence for extra neuronal roles [3], which include liver, heart and metabolism [3, 4, 5, 6, 7, 8, 9, 10, 11]. The changing natural history of treated SMA patients suggests a role for multisystemic SMA management [4]. In line with this, we recently reported fatty liver in SMA patients and steatosis in SMA patient–specific induced pluripotent stem cell–derived hepatocyte‐like cells (iHeps), associated with decreased expression of genes implicated in liver function [5]. Hepatic dysfunction is caused by hepatocyte‐intrinsic SMN deficiency [5], suggesting that SMA treatment needs to address liver as well as neuromuscular pathology. Isogenic carrier iHeps also exhibited steatosis and reduced transcription of key genes implicated in hepatic function [5].

If SMA carriers are susceptible to liver disease, potential clinical implications include: (1) broadening the SMA clinical spectrum to include SMA carriers (usually parents of children with SMA) and (2) extension of systematic surveillance and individualized treatments to SMA carriers if clinically indicated.

In this study, we investigated the presence of hepatocyte‐intrinsic lipid metabolic defects in SMA carriers using iHeps from an SMA proband and his asymptomatic parents.

## 2 Methods

### 2.1 Study Approval

Study and IRB exemption approved by the Human Biomedical Research Office, Agency for Science, Technology and Research, Singapore.

### 2.2 CRISPR/Cas9 Editing

GM24468 (SMA Type 2: Son) and 1‐38G (SMA Type 1) iPSCs were CRISPR/Cas9 genome edited to make 2 *SMN1*‐like gene copies, to generate isogenic wild‐type (Iso‐WT) lines as per [5]. Isogenic carriers have been edited to carry only 1 *SMN1*‐like gene copy.

### 2.3 Stem Cell Culture

The SMA patient‐derived hiPSC cell line 1‐38G was provided by Prof. Lee Rubin from Harvard University. GM23720 (WT), GM24468 (SMA Type 2: Son), GM24474 (SMA Carrier: Mother), GM03815 (SMA Carrier: Father) were purchased from Coriell Institute for Medical Research (New Jersey, USA). BJ was from ATCC (Manassas, VA), and H9 hES cells were from WiCell (Madison, Wi). Culture and maintenance were performed as per [5].

### 2.4 Reprogramming of Fibroblast

To create iPSCs from GM03815, which are skin fibroblasts, fibroblasts were reprogrammed following manufacturer's instructions using CytoTune‐iPS 2.0 Sendai Reprogramming Kit (Invitrogen, A16517) to obtain human induced pluripotent stem cells (hiPSCs), which were karyotyped (Figure S1).

### 2.5 Immunostaining of iPSCs

hiPSCs were seeded onto matrigel‐coated 24well plates. Cells were washed with DPBS and fixed in 4% paraformaldehyde (Nacalai Tesque #09154‐85) for 30 min, permeabilized in 0.2% Triton‐X in PBS (Sigma #X100) for 15 min and blocked in 1% BSA in PBS with 0.05% Tween for 1 h at room temperature. Washes were carried out between each step. Cells were incubated with primary antibodies (TRA 1‐60 Miltenyi; Nanog RnD; SOX2 Abcam; SSEA4 Stemcell Technologies; OCT4 Santa Cruz) diluted in blocking buffer and incubated overnight at 4°C (1:100 dilution for all antibodies except OCT4 1:50). Cells were washed thrice. The respective secondary antibodies were diluted 1:500 in blocking buffer and incubated at room temperature, in the dark, for 1 h (Alexa Fluor 488 anti‐rabbit Invitrogen; Alexa Fluor 488 anti‐mouse Invitrogen; Alexa Fluor 555 anti‐goat Invitrogen). DAPI was added together with the secondary antibodies at 1:2000. Cells were washed at least 3 times before visualizing with the EVOS Cell Imaging System, ThermoFisher Scientific.

### 2.6 Induced Hepatocyte (iHep) Differentiation

hiPSCs were plated and cultured for 2 days (~60% confluence) before stepwise differentiation into iHeps [5]. Differentiation was induced with RPMI/B27 medium containing 100 ng/mL Activin A, 3 μM CHIR99021, and 10 μM LY294002 (Days 0–2), followed by 50 ng/mL Activin A (day 3), and 20 ng/mL BMP‐4 plus 10 ng/mL FGF‐10 (Days 6 and 8). From Day 10, hepatocyte culture medium supplemented with 30 ng/mL Oncostatin M and 50 ng/mL HGF (Days 10–22) was used. iHeps were harvested on Day 24.

### 2.7 Oil Red Assay

Cells were fixed with 3.7% paraformaldehyde for 30 min, washed with ultrapure water, and incubated with 60% isopropanol for 5 min. After removing isopropanol, ORO working solution was applied for 15 min at 37°C, followed by washes with ultrapure water. Cells were then incubated in hematoxylin for 1 min, washed, and visualized under a bright‐field microscope (Olympus). Images were processed using ImageJ (NIH). For each sample, three randomly selected microscopic fields were converted to grayscale, thresholded to isolate Oil Red–positive regions, and quantified using the “Analyze Particles” function. Cell numbers were determined independently by a blinded researcher based on hematoxylin staining. Total Oil Red intensity was normalized to cell counts.

### 2.8 Measurement of Oxygen Consumption Rate (OCR) Using Seahorse XFe96 Analyzer

The OCR of iHeps was measured using Agilent Seahorse XFe96 Analyzer. Day 24 iHeps were plated on collagen‐coated 96‐well plates (2.5 × 10^5^ cells/well) and cultured for 4 days. Prior to measurement, cells were incubated in Seahorse XF DMEM supplemented with 25 mM glucose and 1 mM sodium pyruvate. OCR was recorded following sequential injections of 2 μM oligomycin, 1 μM FCCP, and 1 μM rotenone/antimycin A. After the assay, cells were fixed with 4% paraformaldehyde and stained with Hoechst 33342 for high‐content imaging (Perkin Elmer) to normalize OCR values. Data were analyzed using One‐Way ANOVA test with Tukey's multiple comparison test.

### 2.9 MTT Assay

iHeps were reseeded in 96‐well plates (10,000 cells/well) for 24 h before the MTT assay (M2003, Sigma‐Aldrich). Cells were incubated with 90 μL hepatocyte medium containing 10 μL 12 mM MTT at 37°C for 1 h, followed by 100 μL DMSO for another 1 h. Absorbance was measured at 595 nm using a Synergy H1 microplate reader.

### 2.10 Western Blot

Cells were lysed in Pierce RIPA buffer (Thermo Fisher), and clarified lysates were quantified by BCA assay (Sigma‐Aldrich). Equal protein amounts were denatured (95°C, 5 min), separated by SDS‐PAGE, and transferred to nitrocellulose membranes (Bio‐Rad) at 4°C for 1 h. Membranes were blocked (2.5% milk, TBS‐T) and probed overnight at 4°C with Beta‐actin (A5441, Sigma‐Aldrich) or SMN antibodies in TBS with 5% BSA, followed by HRP‐conjugated secondary antibody (32,430, Thermo Fisher) for 1 h. Detection was performed using SuperSignal West Femto substrate (Thermo Fisher) on an iBright Imaging system (Invitrogen).

### 2.11 Rt‐PCR

RNA was extracted using TRIzol Reagent (Invitrogen), and 1000 ng RNA was reverse transcribed with the High‐Capacity cDNA Reverse Transcription Kit (Applied Biosystems) under the following conditions: 25°C for 10 min, 37°C for 120 min, and 85°C for 5 min. RT‐qPCR was performed on a 7500 Real‐Time PCR System (Applied Biosystems) using SYBR Green probes and Universal PCR Master Mix, with Bata‐actin as the housekeeping gene. Relative mRNA expression levels were calculated using the ΔΔCT method.

### 2.12 Proteomics

Cell pellets harvested on Day 24 were analyzed by tandem mass spectrometry using an Orbitrap Fusion Eclipse system (Thermo Fisher Scientific) operated in data‐dependent acquisition mode. Proteome comparisons between groups were presented as volcano plots. The plots' *x*‐axis corresponded to log_2_ fold change (log_2_ FC) while the *y*‐axis represented the –log_10_ *p* value. Significance cut‐offs representing FC of at least 1.2 and *p* value < 0.01 were indicated by dotted lines on the plots. Table S1 contained the complete list of the significantly‐different proteins.

### 2.13 Risdiplam Treatment of iHeps

On Day 24, 1 μM Risdiplam (#T16757) (TargetMol, USA) reconstituted in 100% ethanol, was added to the iHep maintenance media or differentiation media for 7 days.

## 3 Results

### 3.1 SMA and SMA Carrier‐Derived iHeps Show Increased Lipid Accumulation Which Is Reduced by Risdiplam Treatment

**FIGURE 1:**
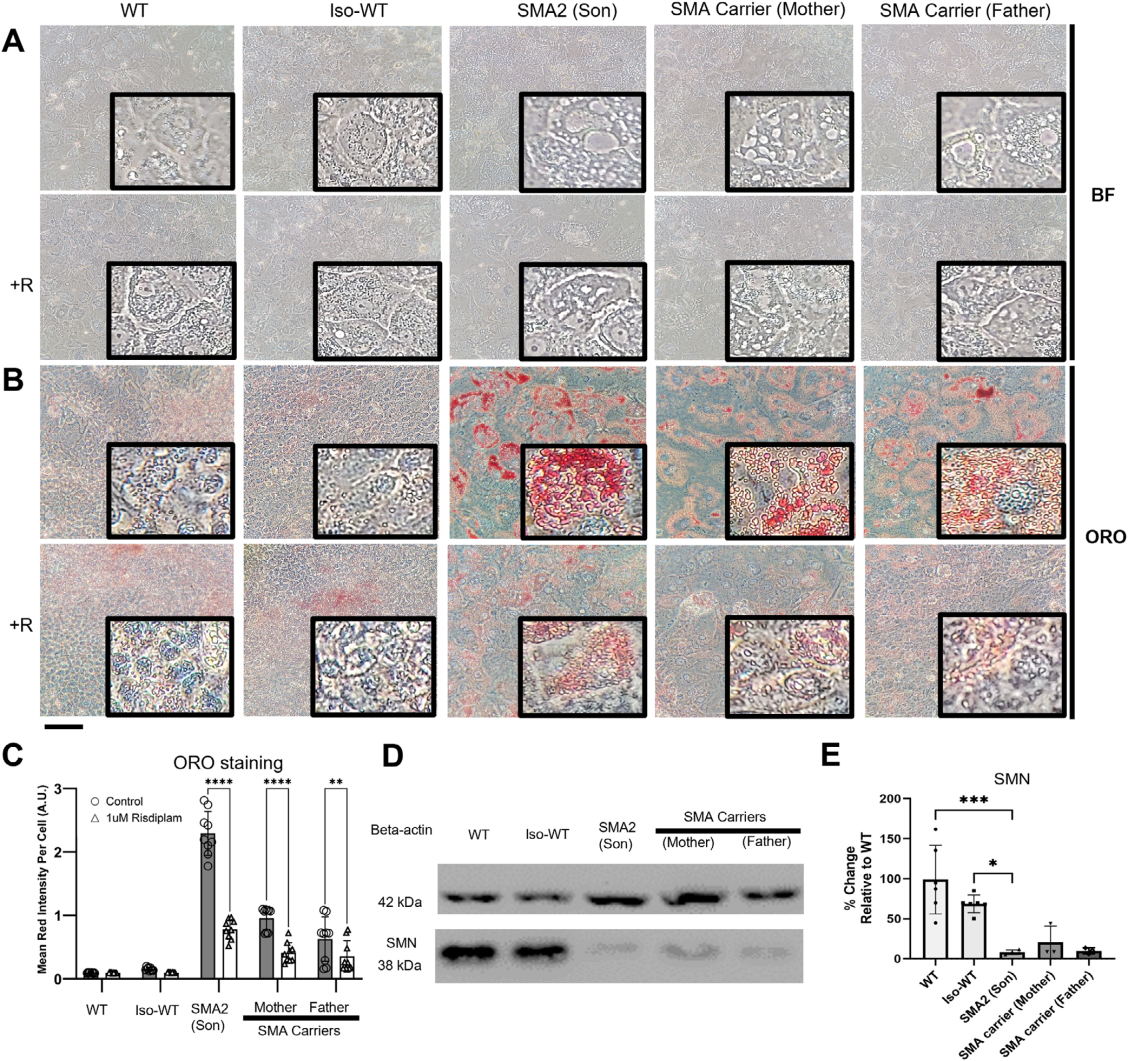
Risdiplam treatment partially reverses steatosis and oil red accumulation in Day 24 SMA type 2 (SMA2) and SMA carrier iHeps. (A) Brightfield microscope images of Day 24 WT, Iso‐WT (Crispr/cas9 edited GM24468 iPSCs to replenish 2 SMN1‐like copies), SMA2 (GM24468) and SMA carrier iHeps GM24474 (mother); GM03815 (father). (B) Brightfield microscope images of Oil Red O (ORO) stained Day 24 WT, Iso‐WT, SMA2 and SMA carrier iHeps. (A‐B) Boxed portions represent zoomed‐in segments of the original image to showcase more clearly. +R: With treatment of 1uM Risdiplam. Ethanol was used as vehicle control. Scale bar = 50 μm. (C) Mean red intensity per cell as compared to control. (*n* = 9 per group) (D) Representative western blot image of SMN expression from three independent runs. (E) Image J analysis of (D) for SMN expression normalized to beta‐Actin housekeeping protein (WT *n* = 6, Iso‐WT *n* = 6, SMA2 *n* = 4, SMA carrier (Father) *n* = 3, SMA carrier (Mother) *n* = 3) (C & E) Data were analyzed using One‐Way ANOVA test with Tukey's multiple comparison test. Data are presented as mean (±SD). *p value < 0.05; **p value < 0.01; ***p value < 0.001; ****p value < 0.0001.

iHeps derived from a child with SMA Type 2 and his carrier parents had increased numbers of small and large vacuoles (Figure 1A) that stained with Oil Red O (ORO) (Figure 1B). SMA and carrier‐ derived iHeps had 15‐fold and 5‐fold increase in lipid accumulation respectively, compared with WT and Iso‐WT generated from the child (Figure 1C). On Western Blot (Figure 1D, Figure S2), SMA and carrier‐derived iHeps expressed approximately 10% of WT and isogenic WT SMN expression (Figure 1E). Risdiplam treatment effectively reduced lipid accumulation in SMA‐derived iHeps by 65.9%, in father carrier–derived iHeps by 43.6%, and in mother carrier–derived iHeps by 56.9%, with all groups showing reductions of more than 40% (Figure 1B,C).

### 3.2 SMA and Isogenic Carrier iHeps Have Distinct Proteome Alterations Implicating Lipid Metabolism

**FIGURE 2:**
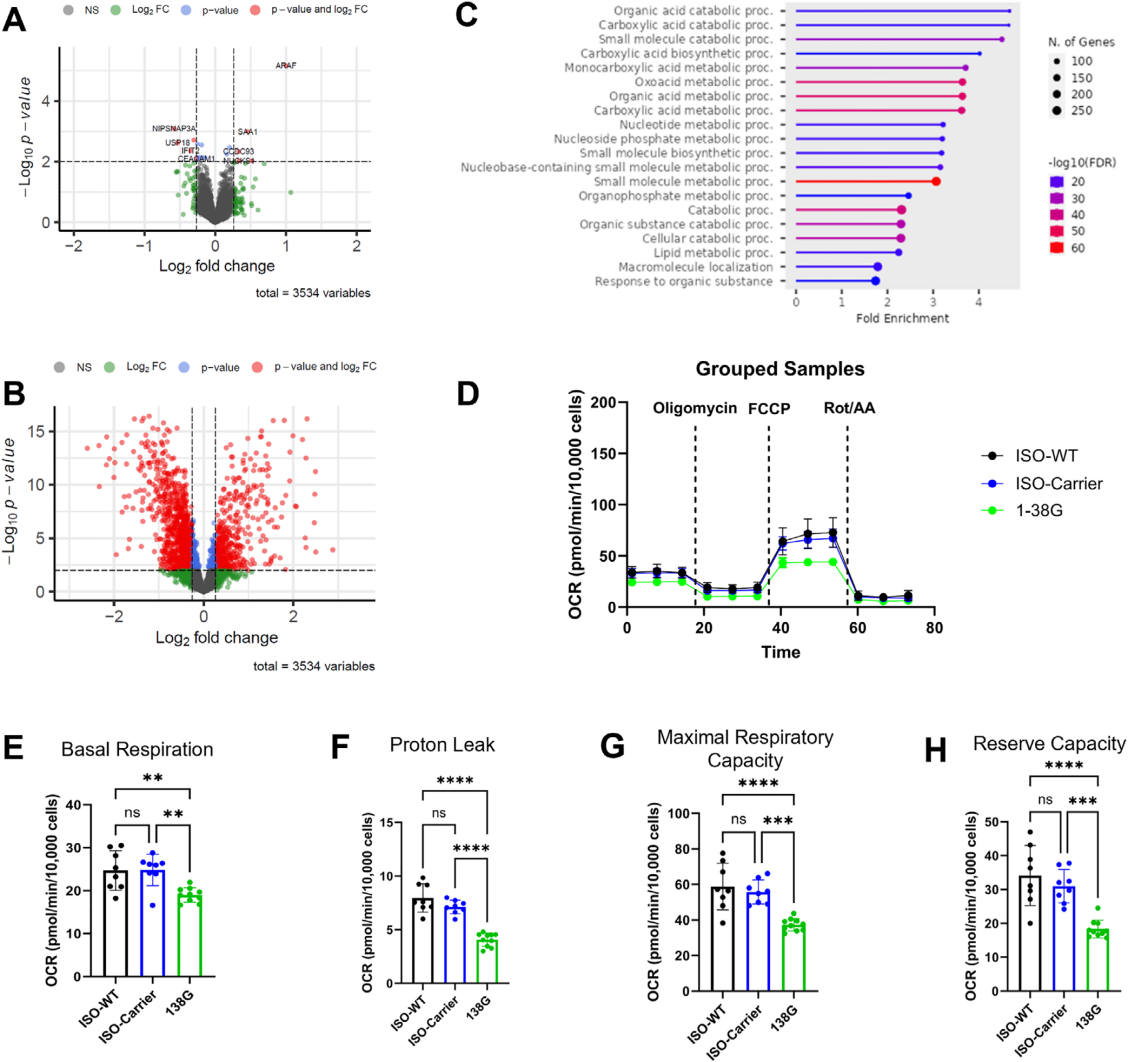
SMA carrier iHeps are more similar to Iso‐WT as compared to SMA iHeps. Proteome comparisons of the (A) iHeps between Iso‐Carrier and Iso‐WT cells and the (B) iHeps between SMA type 1 (SMA1: 1‐38G) and Iso‐WT cells. Gray dots were non‐significant proteins (NS), green dots represented proteins that passed only the log_2_ FC cut‐off, and blue dots were proteins that passed only the –log_10_ *p* value cut‐off. Red dots represented proteins that passed both cut‐offs, yielding 10 and 1281 significantly‐different proteins for (A, B), respectively. (C) showed the top 20 pathways based on Gene Ontology terms enrichment from ShinyGO analyses of the significantly‐different proteins in (B). Each horizontal line represented the fold enrichment of the term shown to the left and each dot indicated the size (number) of significantly‐different proteins containing the enriched terms. (D) Measurements of OCR between ISO‐WT, ISO‐Carrier and SMA Type 1 (SMA1) iHeps (error bars = SEM, ISO‐WT *n* = 8, ISO‐Carrier *n* = 9, 138G *n* = 10), with analysis of (E) Basal respiration, (F) proton leak, (G) maximal respiratory capacity, (H) mitochondrial reserve capacity. (E—H) Data are presented as mean (±SD). *p value < 0.05; **p value < 0.01; ***p value < 0.001; ****p value < 0.0001.

We showed using isogenic lines that SMA iHeps had distinct proteome alterations in lipid metabolism pathways (Figure 2A,B, Table S1). SMA isogenic carrier iHeps also had dysregulated expression of proteins implicated in lipid metabolism, such as A‐Raf Proto‐Oncogene, Serine/Threonine Kinase (ARAF) [12]; Protein NipSnap homolog 3A (NIPSNAP3A) [13], serum amyloid A1 (SAA1) [14], ubiquitin specific peptidase 18 (USP18) [15], Coiled‐coil domain‐containing protein 93 (CCDC93) [16], Carcinoembryonic antigen‐related cell adhesion molecule 1 (CEACAM1) [17] (Figure 2C). Mitochondrial oxygen consumption rate (OCR), though limited in replicates, offer preliminary evidence of impaired mitochondrial bioenergetics only in SMA iHeps (Figure 2D–H).

### 3.3 SMA and SMA Carrier‐Derived iHeps Show Dysregulation of Genes Implicated in Lipid Metabolism and Hepatic Function

**FIGURE 3:**
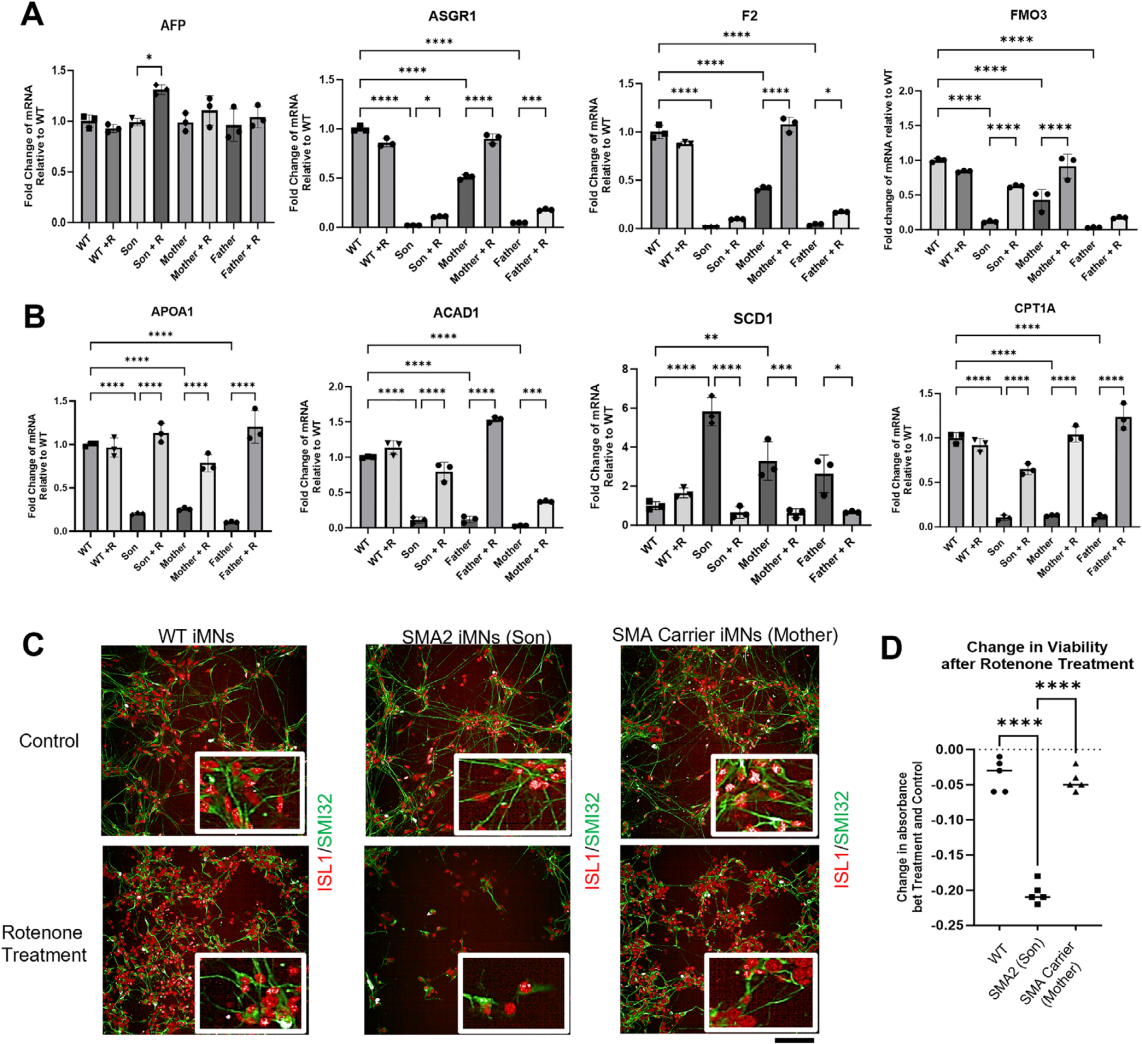
SMA2 and SMA carrier iHeps show dysregulation of genes implicated in hepatocyte function, and lipid transport and metabolism; SMA2 patient iMNs are more susceptible to stress treatments. (A, B) RT‐qPCR of (A) hepatocyte function biomarkers and (B) lipid transport and lipid metabolism genes in SMA2 and SMA carrier iHeps at day 24. Unless specifically indicated that outliers were removed, analysis of data from three independent experiments included three samples (*n* = 3) each for WT (BJ), SMA2 and SMA carrier conditions. +R: With treatment of Risdiplam. (C) Confocal images of Day 28 iMNs stained with SMI32 (green) and ISL1 (red) before and after 200 nM rotenone for 24 h. (D) Exploratory MTT assay showed the difference in cellular metabolic activity between control (no treatment) and stress conditions in SMA patient, carrier and WT control iMN. WT: H9; GM 24468 Son: SMA Type II; GM24474 Mother: SMA Carrier. Data generated from one experiment (*n* = 1), each with five technical replicates. Data were analyzed using One‐Way ANOVA test with Tukey's multiple comparison test. Data are presented as mean (±SD). *p value < 0.05; **p value < 0.01; ***p value < 0.001; ****p value < 0.0001.

We hypothesized that genes relating to lipid metabolism and hepatic function would also be affected in carrier‐derived induced iHeps from the mother and father (Figure 3). Genes associated with hepatic function, including glycoprotein homeostasis (ASGR1), clotting (F2) and drug metabolism (FMO3) [5] showed reduced transcription in SMA and carrier‐derived iHeps (Figure 3A). Similar findings were found for genes involved in lipid transport: albumin (ALB) and apolipoprotein A1 (APOA1), and fatty acid beta‐oxidation (FAO): carnitine palmitoyltransferase 1A (CPT1A) and acyl‐CoA dehydrogenase medium chain (ACAD1), while the converse was true for stearoyl‐CoA desaturase (SCD1), the rate‐limiting enzyme for de novo lipogenesis [5] (Figure 3B). Risdiplam reversed changes in SMA and carrier iHeps and did not cause changes to WT iHeps. As part of an exploratory analysis, SMA carrier‐derived induced motor neurons (iMNs) did not exhibit reduced viability with rotenone stress, whereas the converse was true for SMA iMNs (Figure 3C,D).

## 4 Discussion

In this report, we demonstrated iHep lipid droplet accumulation and proteotranscriptomic dysregulation, partially reversed by risdiplam, in a SMA proband and his parents. This supports our previous findings of steatosis in isogenic carrier iHeps [5], which are biologically dissimilar to the parents in this study, who are in turn biologically dissimilar to each other. Since they are unrelated individuals, the observed dysfunction in their hepatocyte‐like cells is SMA related and not caused by an unrelated shared gene. Isogenic WT controls did not show steatosis, suggesting the phenotype tracks with SMN dosage rather than the polygenic background. All differentiations were performed in parallel using standardized protocols, and WT iHeps consistently lacked fat accumulation across differentiations, excluding variability in iPSC reprogramming or iHep differentiation as a cause. Unlike hepatic steatosis in SMA patients [5], we speculate that the mechanism of hepatic steatosis in SMA carriers is not related to defects in mitochondrial bioenergetics. Further studies will be required to experimentally validate this possibility. Carrier‐derived iMNs have normal viability, as expected from normal neuromuscular clinical phenotype of SMA carriers. These observations provide evidence to further support the increasing body of evidence of extra‐neuronal manifestations of SMA [3, 4, 5, 6, 7, 8, 9, 10, 11]. Some cell types may be more affected quantitatively by the loss of SMN than others [6]. One other study has suggested that SMA carriers may exhibit subclinical pathology, specifically, mitochondrial defects in skin fibroblasts from SMA carriers [7]. If clinically symptomatic, SMA carriers should be termed “SMA heterozygotes”, instead of “carriers”, because the latter implies there are no clinical manifestations. This could potentially have profound impacts on understanding the spectrum of SMA disease and its management.

A limitation of our study is the small number of biological samples for SMA carriers. The immature iHeps used in this study may lack full metabolic maturity. Additionally, this study did not include relevant in vivo assessments, such as clinical liver imaging, bloodwork including traditional liver function tests, or functional outcomes. We acknowledge that lipid accumulation does not necessarily equate to clinically significant steatosis, especially in vitro. The proband and carrier parents did not have their liver phenotypes recorded by the company from which their cells were purchased. However, we have previously shown that iHeps steatosis correlate with sonographic evidence of fatty liver in SMA [5].

Next steps would include exploring the prevalence and clinical significance of fatty liver in SMA cohorts and SMA carriers, with longitudinal imaging and lipid panels to confirm fatty liver and to monitor whether this eventually leads to liver inflammation, cirrhosis, or impaired liver function, and correlating underlying molecular mechanisms with clinical phenotypes using liver biopsies or mouse models.

## Author Contributions

Designing research studies: Crystal J.J. Yeo. Providing reagents, conducting experiments, acquiring data, and/or analyzing data: Crystal J.J. Yeo, Damien Meng Kiat Leow, Lingyu Sun, Loo Chien Wang, Michelle Yating Eio, Hiromi W.L. Koh, Zi Jian Khong, Gunaseelan Narayanan, Aloysius Kai Soon Teo, Richard Giadone, Radoslaw M. Sobota, Aloysius Kai Soon Teo, Shi Yan Ng, Wei Yi Ong, Lee L. Rubin, and Basil T. Darras. Writing the manuscript: Crystal J.J. Yeo, Lingyu Sun, and Damien Meng Kiat Leow.

## Funding

This work was supported by the Agency for Science, Technology and Research (C210112024) and National Neuroscience Institute (IRNMR21CPGJJ).

## Ethics Statement

We confirm that we have read the Journal's position on issues involved in ethical publication and affirm that this report is consistent with those guidelines.

## Conflicts of Interest

The institution of Basil T. Darras has received personal compensation in the range of $500–$4999 for serving as a Consultant for Amicus. Basil T. Darras has received personal compensation in the range of $0–$499 for serving on a Scientific Advisory or Data Safety Monitoring board for Amicus. The institution of Basil T. Darras has received research support from the National Institutes of Health/National Institute of Neurological Disorders and Stroke. The institution of Basil T. Darras has received research support from the Slaney Family Fund for SMA. The institution of Basil T. Darras has received research support from the Spinal Muscular Atrophy Foundation. The institution of Basil T. Darras has received research support from CureSMA. The institution of Basil T. Darras has received research support from the Working on Walking Fund. The institution of Basil T. Darras has received research support from CHERISH, CS2/CS12. The institution of Basil T. Darras has received research support from Biogen for CS11. The institution of Basil T. Darras has received research support from AveXis. The institution of Basil T. Darras has received research support from Sarepta Pharmaceuticals. The institution of Basil T. Darras has received research support from PTC Therapeutics. The institution of Basil T. Darras has received research support from Roche. The institution of Basil T. Darras has received research support from Santhera. The institution of Basil T. Darras has received research support from Scholar Rock. The institution of Basil T. Darras has received research support from Fibrogen. The institution of Basil T. Darras has received research support from Summit. Basil T. Darras has received publishing royalties from a publication relating to health care. The other authors declare no conflicts of interest.

## Supporting information


**Figure S1:** Quality control of GM03815 iPSCs. (A–C) Immunofluorescence staining of GM03815 iPSCs. Scale bar: 300 μm. (A) NANOG and SSEA4. (B) TRA160 and SOX2 (C) OCT4 (D) Karyotyping of chromosomes from GM03815 iPSCs.
**Figure S2:** Western blot image of SMN expression. The whole western blot image of (A) beta‐actin housekeeping protein and (B) SMN expression shown in Figure 1.

**Table S1:** Comparison of differentially‐expressed proteins of iHeps carrier (138G) versus isogeneic control (WT) Proteomics analysis of iHeps carrier and isogeneic control was carried out and yielded 3534 overlapping proteins between the two conditions. Using R script, proteins with logFC > 1.2 and log *p* value < 0.01 were accepted as significant cutoffs for differential expression in the carrier compared to WT. This table lists all the proteins that were identified from the proteomics comparison.

## Data Availability

The mass spectrometry data have been deposited in the ProteomeXchange Consortium (http://proteomecentral.proteomexchange.org) via the Japan Proteome Standard Repository partner (https://repository.jpostdb.org/) with the dataset identifier PXD045401.
