# Supplementary material for: Liver Steatosis in Induced Hepatocytes From Carriers of Spinal Muscular Atrophy
Source: Muscle Nerve. 2026 Jan 22;73(4):675–82. doi: 10.1002/mus.70111 (PMC12969962; doi:10.1002/mus.70111)
Supplement: Supplementary file 1 — Figure S1: Quality control of GM03815 iPSCs. (A–C) Immunofluorescence staining of GM03815 iPSCs. Scale bar: 300 μm. (A) NANOG and SSEA4. (B) TRA160 and SOX2 (C) OCT4 (D) Karyotyping of chromosomes from GM03815 iPSCs. Figure S2: Western blot image of SMN expression. The whole western blot image of (A) beta‐actin housekeeping protein and (B) SMN expression shown in Figure 1. [file MUS-73-675-s002.docx]

**Supplementary Figures:**

**
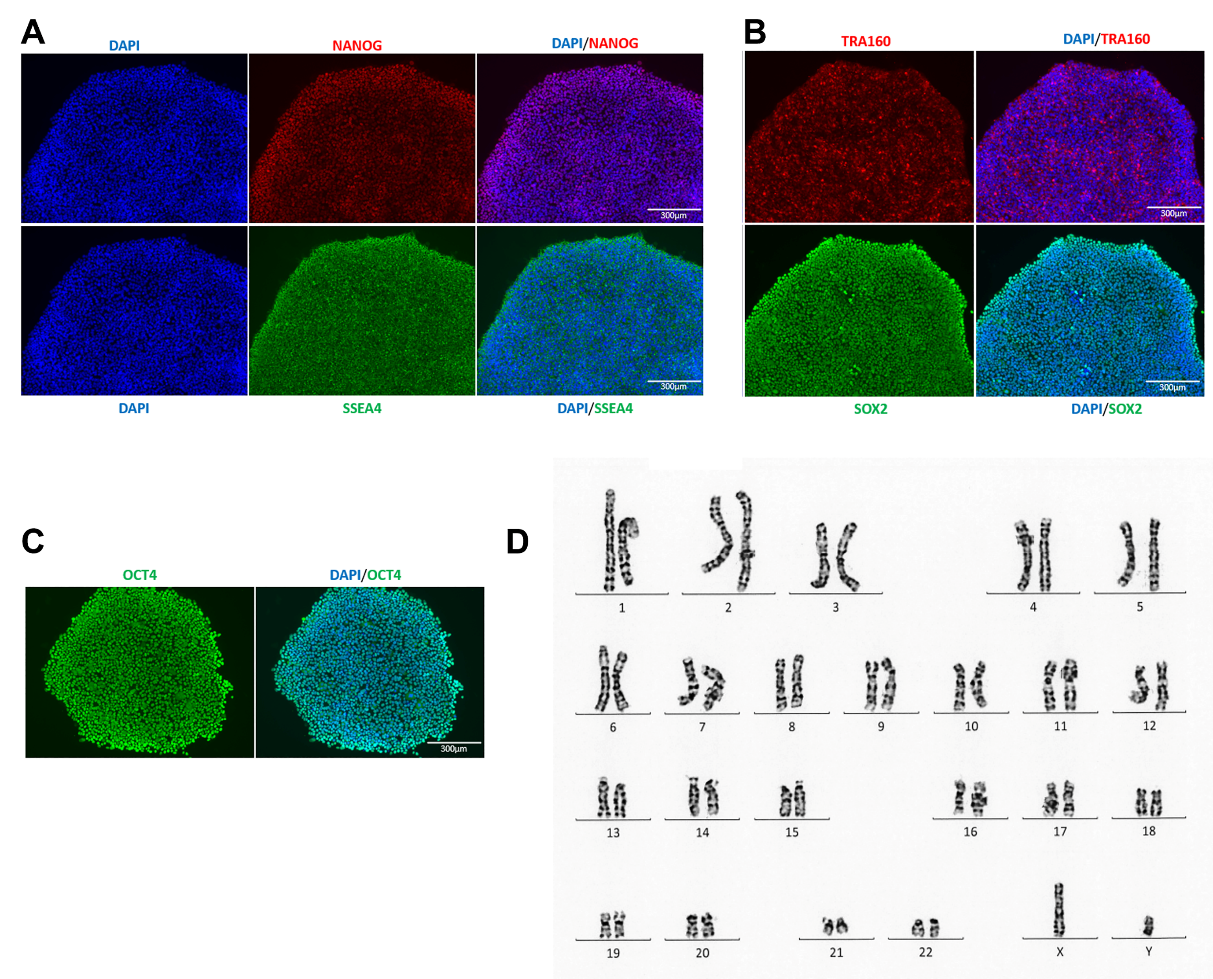
**

**Supplementary Figure 1: Quality control of GM03815 iPSCs. (A-C)** Immunofluorescence staining of GM03815 iPSCs. Scale bar: 300µm. **(A)** NANOG and SSEA4. **(B)** TRA160 and SOX2 **(C)** OCT4 **(D)** Karyotyping of chromosomes from GM03815 iPSCs.

**
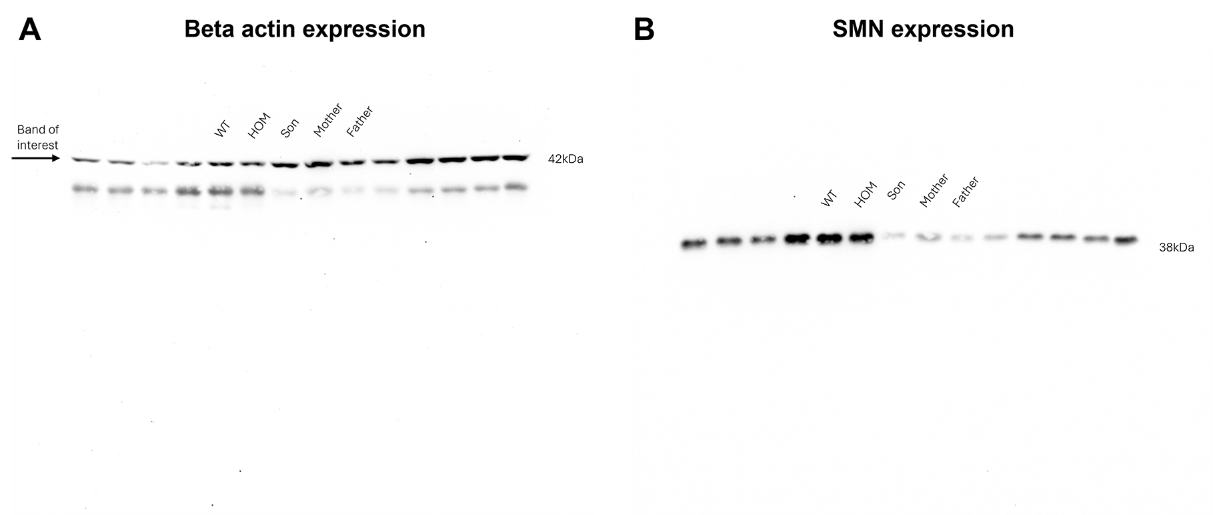
**

**Supplementary Figure 2: Western blot image of SMN expression.** The whole western blot image of **(A)** beta-actin housekeeping protein and **(B)** SMN expression shown in Figure 1.
